# Prognostic risk of immune-associated signature in the microenvironment of brain gliomas

**DOI:** 10.3389/fgene.2023.1208651

**Published:** 2023-10-06

**Authors:** Yaling Tao, Junqi Zhu, Xiaoling Yu, Huaiwei Cong, Jinpeng Li, Ting Cai, Qian Chen

**Affiliations:** ^1^ Ningbo No 2 Hospital, Ningbo, China; ^2^ Ningbo Institute of Life and Health Industry, University of Chinese Academy of Sciences, Ningbo, China; ^3^ Thorgene Co., Ltd., Beijing, China; ^4^ Ningbo Hangzhou Bay Hospital, Ningbo, China

**Keywords:** brain glioma, prognosis, risk score, immune-related genes, immune cells, tumor microenvironment

## Abstract

Understanding the key factors in the tumor microenvironment (TME) that affect the prognosis of gliomas is crucial. In this study, we sought to uncover the prognostic significance of immune cells and immune-related genes in the TME of gliomas. We incorporated data of 970 glioma patient samples from the Chinese Glioma Genome Atlas (CGGA) database as the training set, and an additional set of 666 samples from The Cancer Genome Atlas (TCGA) database served as the validation set. From our analysis, we identified 21 immune-related differentially expressed genes (DEGs) in the TME, which holds implications for glioma prognosis. Based on these genes, we constructed a prognostic risk model on the 21 genes. The prognostic risk model demonstrated robust performance with an area under the curve (AUC) value of 0.848. Notably, the risk score derived from the model emerged as an independent prognostic factor of gliomas, with high risk scores indicative of an unfavorable prognosis. Furthermore, we observed that high infiltration levels of certain immune cells, namely, activated dendritic cells, M0 macrophages, M2 macrophages, and regulatory T cells (Tregs), correlated with an unfavorable glioma prognosis. In conclusion, our findings suggested that the TME of gliomas harbored a distinct immune-associated signature, comprising 21 immune-related genes and specific immune cells. These elements significantly influence the prognosis and present potential as novel indicators in the clinical assessment of glioma patient outcomes.

## 1 Introduction

Glioma is the most common and aggressive primary brain malignancy in adults, originating from cancerous glial cells in the brain and spinal cord. It accounts for more than 30% of the total intracranial tumors and 2% of adult cancers (Stupp et al., 2009; Gilbert et al., 2014; Hu et al., 2017; Zhang et al., 2018). According to the current research, there are three major signaling pathway alterations that can elicit glioma formation, namely, the growth factor signaling pathway of the receptor tyrosine kinase (RTK) genes, the phosphatidylinositol 3-kinase (PI3K) pathway, and the p53 tumor suppressor pathways (Furnari et al., 2007). Many treatment options are available for gliomas, including surgery, radiotherapy, systemic therapy (chemotherapy and targeted therapy), supportive therapy, and temozolomide combined with chemotherapy; however, the overall prognosis remains poor and long-term survival rates are low (Stupp et al., 2005; Tan AC. et al., 2020). Therefore, it is crucial to determine effective and accurate prognostic indicators for gliomas.

The tumor microenvironment (TME) is an extremely heterogeneous system. Immune cells are the critical non-tumor components of the TME that are involved in the generation, growth, and progression of cancer (Iyengar et al., 2016; Hoy et al., 2017; Wu et al., 2019). Identifying immune conditions and immune-related genes in the TME that affect prognosis is significant. Yoshihara K. *et al* designed an algorithm called ESTIMATE to calculate immune and stromal scores to predict the level of cell infiltration by analyzing the expression profiles of specific genes in immune and stromal cells (Yoshihara et al., 2013). The prognostic value of immune-related genes in the TME has been demonstrated in several trials (Galon et al., 2012; Pagès et al., 2018; Fakih et al., 2019; Yang et al., 2020). Jia D. *et al* identified the genes such as *IL-13RA2*, *CCL2*, *IL-6*, *TLR2*, *COL1A2*, *TIMP1*, *THBS1*, and *SERPINE1* in the TME of glioma patients associated with poor prognosis based on the ESTIMATE algorithm (Jia et al., 2018). However, single genes as prognostic indicators often tend to lead to less accurate predictions of prognostic risk due to individual differences.

To avoid individual differences, researchers have combined multiple genes to construct prognostic risk models. Cheng W. *et al.* used eight immune genes with the greatest prognostic value, namely, *FOXO3*, *IL-6*, *IL-10*, *ZBTB16*, *CCL18*, *AIMP1*, *FCGR2B*, and *MMP9*, to develop an immune-related risk signature for gliomas that could independently distinguish high-risk patients (Yi et al., 2019). Tan Y.Q. *et al* constructed a prognostic model to predict the outcomes of low-grade gliomas based on six genes, namely, *CD163*, *FPR3*, *LPAR5*, *P2ry12*, *PLAUR*, and *SIGLEC1* (Tan YQ. et al., 2020). However, the important correlation between glioma prognosis and immune-related genes and immune cells is still unclear. In this study, we identified 21 immune-related genes differentially expressed in the TME that affect the prognosis of gliomas. We evaluated the prognostic impact of the risk score from these 21 immune-related genes and the correlation between immune cells and prognosis in the TME.

This study intends to construct a highly accurate and sensitive prognostic risk model based on immune-related genes, and provide independent prognostic factors for the clinical prognosis assessment of glioma patients, so as to elucidate the critical role of immune cells and related genes in the TME and its impact on the prognosis of patients with gliomas.

## 2 Materials and methods

### 2.1 Data collection

The transcriptomic data and clinical data of 970 glioma patients were downloaded from the Chinese Glioma Genome Atlas (CGGA) database (http://www.cgga.org.cn/) as the training cohort. For the validation cohort, transcriptomic data and the clinical data of 666 glioma patients were obtained from The Cancer Genome Atlas (TCGA) (https://portal.gdc.cancer.gov/). Samples with gene expression 0 were excluded. A total of 1811 immune-related genes were downloaded from the Immunology Database and Analysis Portal (IMMPORT; https://www.immport.org/).

### 2.2 Calculation of immune score and stromal score

Based on gene expression data, the ESTIMATE algorithm was used to calculate the stromal and immune scores for each tumor sample, predicting the levels of stromal and immune cells. The stromal score and immune score calculated by this algorithm helped quantify the stromal and immune cells in the tumor. The glioma patients were then divided into high- and low-stromal score subgroups and high- and low-immune score subgroups based on the median stromal score and immune score.

### 2.3 Recognition of differentially expressed genes

The R package limma was used to identify DEGs between high- and low-stromal score subgroups, as well as high and low immune scores. The cut-off criteria were set as |fold change| greater than 2 and *p* value less than 0.05. To reduce the false positive rate, the eBayes test was used to adjust the *p* value. The heatmaps and volcano plots of the DEGs were displayed using the ComplexHeatmap and “ggplot2” packages in R, respectively. Shared DEGs were analyzed using immune gene sets, DEGs of the stromal score group and the immune score group. A Venn diagram to display the crossover genes was constructed using the R package “venn”.

### 2.4 GO and KEGG pathway enrichment analyses

The Gene Ontology (GO) and Kyoto Encyclopedia of Genes and Genomes (KEGG) enrichment analyses were used to identify the characteristic biological attributes and functional attributes of DEGs, respectively. GO and KEGG analyses were performed using the R package “clusterProfiler” and “org.Hs.eg.db”. Enrichment results were visualized using the R package “ggplot2”, and a *p*-value of less than 0.05 and a false discovery rate (FDR) of less than 0.05 were considered as the criteria for significant enrichment.

### 2.5 Development of an immune gene-related prognostic risk model

The shared DEGs with *p*-values less than 0.05 were screened by univariate Cox analysis. The screened immune-related DEGs were further selected by Lasso regression with the R package “SIS” to identify the final variables used to construct the Cox model. A multivariate Cox regression risk model was then constructed using the final 21 DEGs. The results of the multivariate Cox analysis were presented with the R package “forestplot”. The risk score of the Cox model was calculated based on a linear combination of coefficients and gene expression levels. Its formula is as follows:

$$\text{Risk score}=\left({\text{Coef}}_{1}*{\mathrm{Exp}}_{1}\right)+\left({\text{Coef}}_{2}*{\mathrm{Exp}}_{2}\right)+\ldots +\left({\text{Coef}}_{n}*{\mathrm{Exp}}_{n}\right).$$ (1)

Here, n represents the gene number. Exp represents the gene expression level. coef represents the coefficient value. The median risk score was set as the cut-off value, and all glioma patients were divided into high-risk and low-risk score groups. Receiver operating characteristic (ROC) curves of the multivariate Cox analysis were used to evaluate the performance of the model for the CGGA data and TCGA data. The model with an area under the curve (AUC) value of greater than 0.7 was considered a reliable model. Risk curves and survival status scatter plots were drawn using the risk scores and survival status of each patient, with the median risk value taken as the cut-off value, which were used to evaluate the predictive effect of the model on the survival prognosis of patients. The nomogram and calibration curves were generated using the “rms” package in R. The decision curve analysis (DCA) was conducted using the “ggDCA” package, specifically designed for creating decision curves.

### 2.6 Survival analysis

Correlations were analyzed between several key variables and overall survival: the stromal score, the immune score, the expression levels of DEGs, the risk score, and the infiltration level of immune cells. The impact of these variables on the overall survival was calculated using Kaplan–Meier survival curves. For these analyses, two R packages, namely, “survival” and “survminer,” were employed. A *p* value less than 0.05 was considered statistically significant.

### 2.7 Analysis of independent prognostic factors

The difference in the risk score between the different genders, WHO grades, and IDH1 mutation states was compared. The statistical significance of the differences between the two groups was estimated using the Wilcoxon test, and the differences between the three groups were assessed by the Kruskal–Wallis test. Univariate and multivariate Cox regression analyses of age, gender, WHO grade, IDH1 mutation status, and risk score were performed and displayed using the R package forestplot. Factors with a *p* value less than 0.05 were considered to be independent prognostic factors.

### 2.8 CIBERSORT algorithm to assess the level of immune cell infiltration

According to gene expression data, the CIBERSORT algorithm was used to assess the immune infractions in each tumor sample and to provide an estimation of the abundances of each immune cell type in a mixed-cell population. The CIBERSORT algorithm was based on a known reference set that provided gene expression signatures for 22 leukocyte subtypes (LM22). The CIBERSORT algorithm was used to quantify the immune cells between high- and low-risk score groups. The results of inferred scores for immune cell populations were considered accurate at a threshold of *p* value 0.05. The distribution of immune cells in the two groups was shown using the ggpubr package. The univariate Cox analysis was performed using the plyr package and visualized using the forestplot package.

### 2.9 Statistical analysis

In this study, all statistical computations and figures were performed using R software (version 3.6.3). The Wilcoxon rank-sum test was used as a non-parametric test for comparison between the two groups, and the Kruskal–Wallis test was used to estimate the differences between three groups. The Wald test was used in the univariate and multivariate Cox analyses. The log-rank test was used to generate *p*-values in Kaplan–Meier survival analysis. A *p* value less than 0.05 was considered to be statistically significant.

## 3 Results

### 3.1 Immune conditions are significantly associated with overall survival of patients with gliomas

**FIGURE 1:**
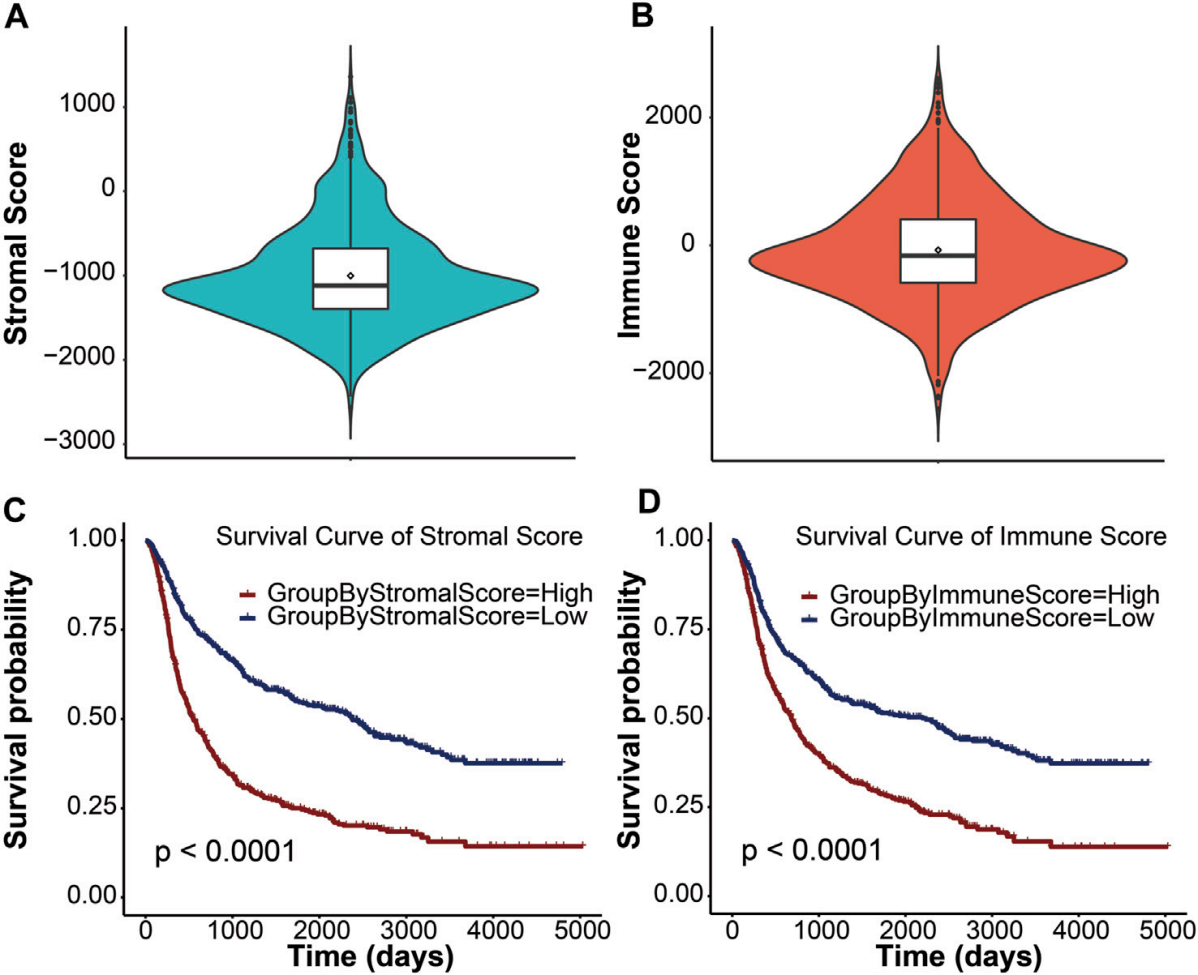
Immune conditions are significantly associated with overall survival of patients with gliomas. Distribution of stromal scores **(A)** and immune scores **(B)** for glioma patients using the violin plot. The horizontal line in the white box indicated the median. The Kaplan–Meier survival curve displaying the survival duration of glioma patients between high- and low-stromal group **(C)** and high- and low-immune group **(D)**, respectively. Patients were divided into the high-score group (red line) and the low-score group (blue line) according to the median score. A *p* value less than 0.0001 was considered statistically significant.

In this study, we obtained gene expression data and clinical information of glioma patients from the CGGA database for the training cohort and the TCGA database for the validation cohort. After excluding patients with incomplete clinical information and normal controls, 970 and 666 glioma patients with gliomas were included in this study (Table 1). These glioma patients were diagnosed pathologically at ages 8–89 years, with a median age of 44 years. Men accounted for 58.37%, and these patients were at different WHO grades (31.48% WHO II, 35.57% WHO III, and 32.64% WHO IV). To evaluate the abundance of stromal cells and immune cells in the tumor microenvironment, we calculated the stromal score and the immune score of all patients in the training cohort using ESTIMATE. The results showed that the stromal score ranged from −2567.35 to 1358.71 and the immune score ranged from −2557.44 to 2611.02 (Figures 1A, B). We then examined the relationship between stromal/immune scores and the different WHO grades in the CGGA database. The results displayed a positive correlation between stromal/immune scores and WHO stages, indicating a higher prevalence of advanced-grade patients within the high-stromal score and high-immune score groups (Supplementary Tables S1, S2). To investigate the association between the stromal score, immune score, and overall survival, we divided 970 glioma patients into a high-score group and a low-score group and performed a Kaplan–Meier survival analysis between the two groups. The results displayed that the median overall survival of glioma patients with a low stromal score (−1400.96) was longer than those with a high stromal score (−677.93), and the median overall survival of low-immune score (−589.07) patients was longer than that of high-immune score (403.25) patients (*p* <0.0001) (Figures 1C, D). These results indicated that stromal score and immune score could be used as prominent predictors of overall survival in patients with gliomas.

**TABLE 1:** Clinical characteristics of the patients. TCGA, The Cancer Genome Atlas; CGGA, the Chinese Glioma Genome Atlas; WHO, the World Health Organization; tumors were graded I ∼ IV according to the histopathological and clinical criteria established by the World Health Organization. The values in brackets indicate percentages.

	No. of patients (*n* = 1636)%
Database	
TCGA	666 (40.71)
CGGA	970 (59.29)
Age	
Median (range)	44 (8–89)
Gender	
Male	955 (58.37)
Female	681 (41.63)
Grade	
WHO II	515 (31.48)
WHO III	582 (35.57)
WHO IV	534 (32.64)

### 3.2 Identification of differentially expressed immune-related genes in brain gliomas

**FIGURE 2:**
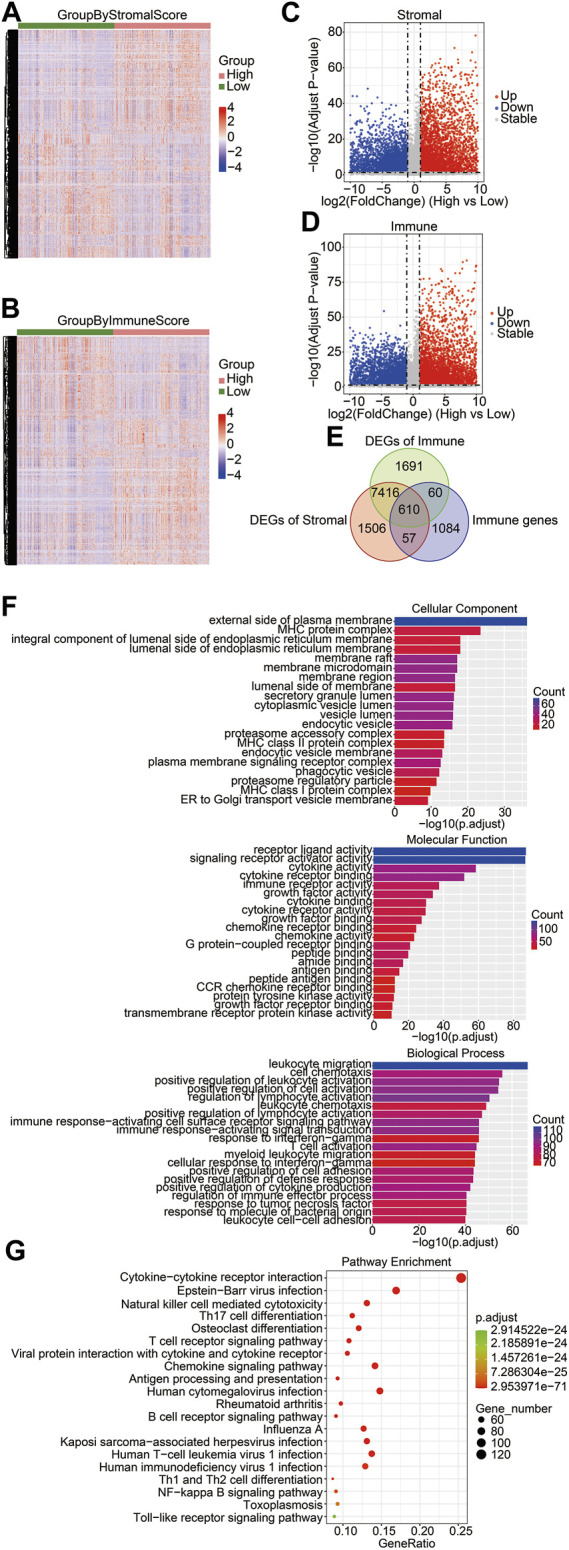
Identification of differentially expressed immune-related genes in brain gliomas. Heatmaps of the DEGs derived from the high-/low-stromal score groups **(A)** and high-/low-immune score **(B)** groups. Green groups represented low-stromal/immune score groups, and red groups were high-stromal/immune score groups. Stromal score groups found a total of 9,586 DEGs, and immune score groups had a total of 9,777 DEGs. Volcano plots of DEGs from the high- and low-stromal score groups **(C)** and the high- and low-immune score groups **(D)**. Red and blue dots represented upregulated and downregulated DEGs, respectively. Gray dots represented no statistically significant genes. **(E)** Venn diagrams showing the shared DEGs in the immune group, stromal group, and immune genes. There were a total of 610 immune-related DEGs. **(F)** GO enrichment analysis of common DEGs. The bar chart exhibited the top 20 significantly enriched signaling pathways, including cellular components (left), molecular functions (right), and biological processes (bottom). **(G)** KEGG function enrichment analysis of the shared DEGs.

To determine key factors in the immune microenvironment that affect prognosis, we analyzed immune-related genes that were differentially expressed in gliomas. First, we compared the DEGs between the high-score group and the low-score group with RNA-seq data of the training cohort, and we identified 9,589 DEGs from the stromal score groups and 9,777 DEGs from the immune score groups. The heatmaps and volcano plots of DEGs are shown in Figures 2A–D. The shared genes between the DEGs of the stromal score groups, the DEGs of immune score groups, and immune genes were analyzed. Among the 1,811 immune-related genes obtained from the ImmPort database, 667 genes were shared with the DEGs of the stromal score groups and 670 were shared with the DEGs of the immune score groups. A total of 610 immune-related DEGs were identified between the stromal and immune score groups (Figure 2E). To explore the functions and enrichment pathways of these immune-related DEGs, GO and KEGG analyses were performed on 610 DEGs. As for GO analysis, we analyzed three subontologies of DEGs: cellular components, molecular functions, and biological processes. We exhibited the top 20 significantly enriched GO terms (Figure 2F). The results indicated that the external side of the plasma membrane and the MHC protein complex were the main enriched cellular components of immune DEGs. The molecular functions of the immune DEGs were primarily focused on receptor–ligand activity and signaling receptor activator activity. The biological process of the immune DEGs mainly included the pathways of leukocyte migration, cell chemotaxis, and positive regulation of leukocyte activation. Subsequently, the results of KEGG pathway enrichment analysis indicated that the DEGs were mainly involved in the pathway of the cytokine–cytokine receptor interaction and the Epstein–Barr virus infection (Figure 2G).

### 3.3 21 Immune-related DEGs significantly related with overall survival

**FIGURE 3:**
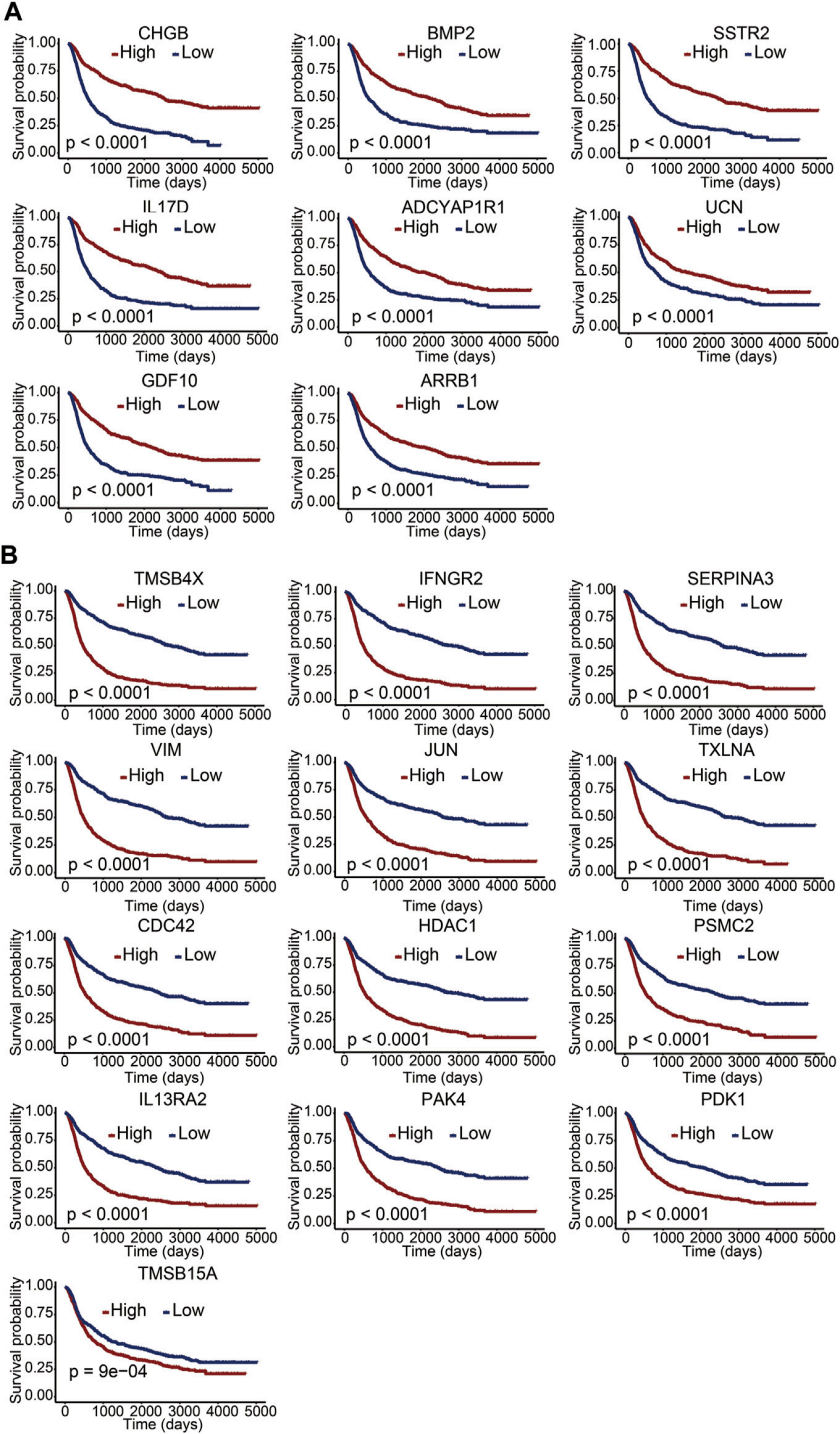
Correlation between immune-related DEGs and overall survival. **(A,B)** Kaplan–Meier survival curves of 21 genes with a *p* value of the log-rank test. A *p* value less than 0.05 represented statistical significance.

To clarify the potential value of immune-related DEGs in the prognosis of glioma patients, we used univariate Cox regression and Lasso regression analysis to screen for the most relevant DEGs. Finally, 21 immune-related DEGs were screened out from the 610 DEGs. A multivariate Cox regression risk model was constructed with the 21 genes (Supplementary Figure S1). We further investigated whether these 21 genes independently affected the overall survival of glioma patients. The Kaplan–Meier survival curves exhibited that the high expression level of *CHGB*, *BMP2*, *SSTR2*, *IL17D*, *ADCYAP1R1*, *UCN*, *GDF10*, and *ARRB1* was significantly correlated with long overall survival (Figure 3A), indicating that these genes are good prognostic genes. Meanwhile, a significant negative association between the expression levels of TMSB4X, IFNGR2, SERPINA3, VIM, JUN, TXLNA, CDC42, HDAC1, PSMC2, IL-13RA2, PAK4, PDK1, and TMSB15A and overall survival is observed in Figure 3B. We then divided the patients into LGG and GBM sets and explored the relationship between the 21 genes and survival rates. The results indicated that the 21-gene signature exhibited significant correlations with the OS in LGG patients, revealing discernible patterns in gene expression, potentially indicative of disease progression (Supplementary Figure S2). However, many genes within our signature lacked a marked impact on OS in GBM subsets (Supplementary Figure S3). This suggested the inherent complexity and heterogeneity of GBM, which might attenuate the influence of individual genes or challenge the signature’s ability to encapsulate the nuanced biology of this subtype.

### 3.4 The risk score derived from the constructed prognostic risk model correlated with overall survival

**FIGURE 4:**
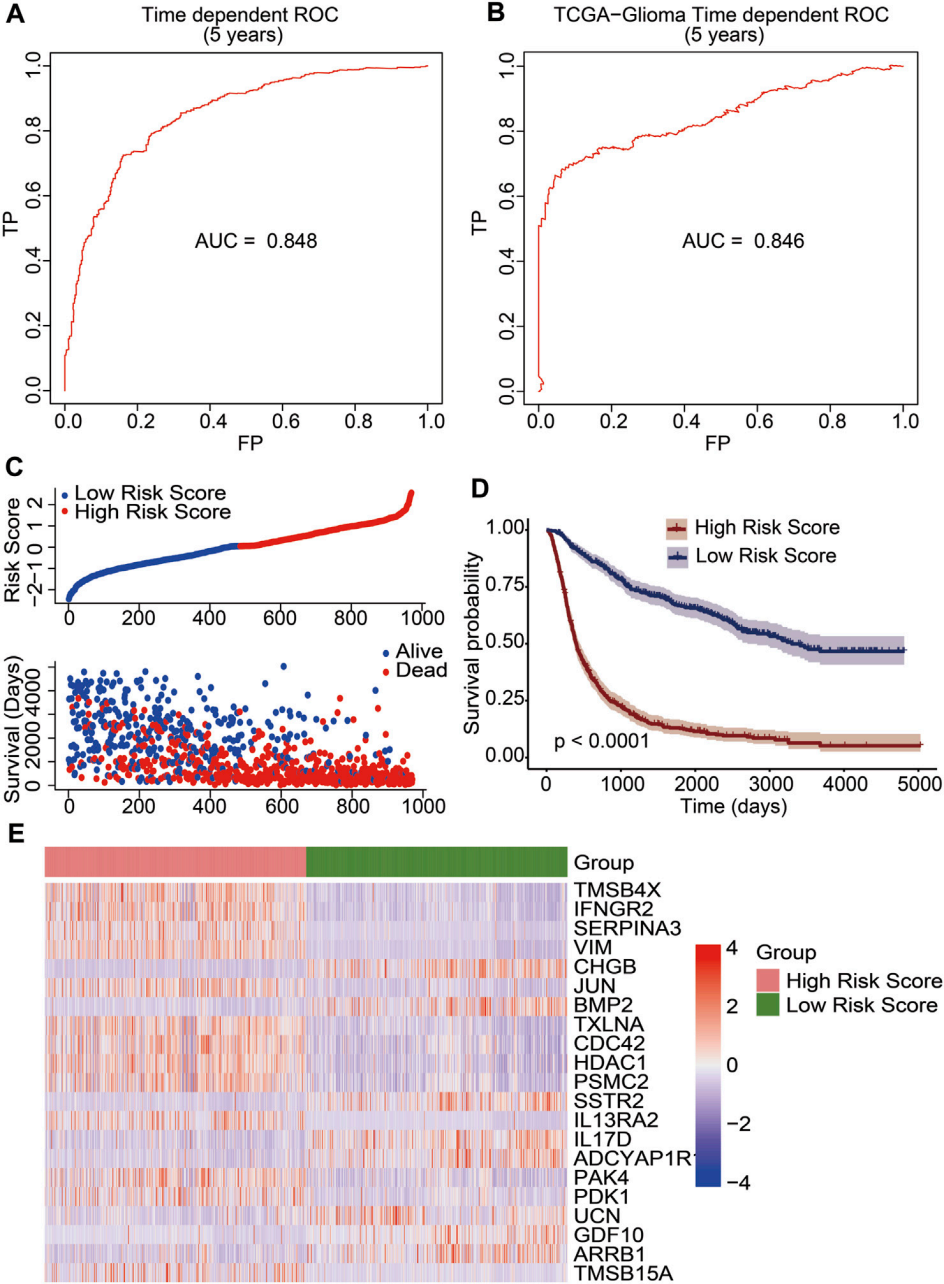
Construction of an immune-related prognostic signature for patients of gliomas. The receiver operating characteristic curve (ROC) of 5-year survival data exhibited risk model credibility for training cohort **(A)** and validation cohort **(B)**. **(C)** Risk score distribution of glioma patients in the CGGA database (top). The blue line represented low risk score, and the red line represented high risk score. Survival status and the duration of patients (bottom). **(D)** Kaplan–Meier survival curves of the high-risk score and low-risk score groups. A *p* value less than 0.05 showed statistical significance. **(E)** Differential expression of 21 immune-related DEGs between the high-risk score group and the low-risk score group.

We developed a prognostic risk model using 21 immune-related DEGs to evaluate the prognosis of glioma patients. The risk scores were calculated based on gene expression levels and their corresponding regression coefficients. The receiver operating characteristic plot for the prognostic model over a 5-year period is shown in Figure 4A, and the area under the curve was 0.848. We then used TCGA data as a validation cohort, and the AUC value of the ROC plot was 0.846 (Figure 4B). These results suggest that the prognostic model based on 21 immune-related DEGs is effective in predicting the prognosis of glioma patients. To further evaluate the model’s efficacy, we divided glioma patients into high-risk and low-risk groups based on their median risk score. The risk score distribution and survival status of glioma patients in the CGGA database showed that the patients in the high-risk score group had more deaths (Figure 4C). Additionally, we observed a significant difference in overall survival between the high-risk and low-risk groups through Kaplan–Meier survival analysis (*p* <0.0001). The low-risk group had a good prognosis, while the high-risk group had a poor prognosis (Figure 4D). Furthermore, we analyzed the expression levels of 21 immune-related genes between the high-risk and low-risk groups. We found that genes associated with a good prognosis (*CHGB*, *BMP2*, *SSTR2*, *IL17D*, *ADCYAP1R1*, *UCN*, *GDF10*, and *ARRB1*) were highly expressed in the low-risk group. Conversely, the genes associated with a poor prognosis (*TMSB4X*, *IFNGR2*, *SERPINA3*, *VIM*, *JUN*, *TXLNA*, *CDC42*, *HDAC1*, *PSMC2*, *IL-13RA2*, *PAK4*, *PDK1*, and *TMSB15A*) were highly expressed in the high-risk group (Figure 4E). Subsequently, we developed a nomogram utilizing the clinicopathological parameters and risk scores to estimate the 3-year and 5-year survival probabilities for glioma patients in the CGGA database (Supplementary Figure S4A) and TCGA database (Supplementary Figure S4B). Furthermore, we conducted a decision curve analysis and analyzed calibration curves to assess the performance of our predictive nomogram. The DCA plots revealed favorable net benefits across the majority of threshold probabilities, specifically indicating optimal threshold probabilities between 0 and 0.92 for the CGGA database (Supplementary Figure S5A) and between 0 and 0.75 for the TCGA database (Supplementary Figure S5C). Additionally, the calibration curves corroborated the concordance between the predicted survival probabilities and the observed outcomes, further substantiating the model’s predictive accuracy (Supplementary Figures S5B, D). The results indicated that the risk score derived from the prognostic model emerged as a molecular indicator of poor prognosis in glioma patients.

### 3.5 The risk score and WHO stage were independent prognostic factors of gliomas

**FIGURE 5:**
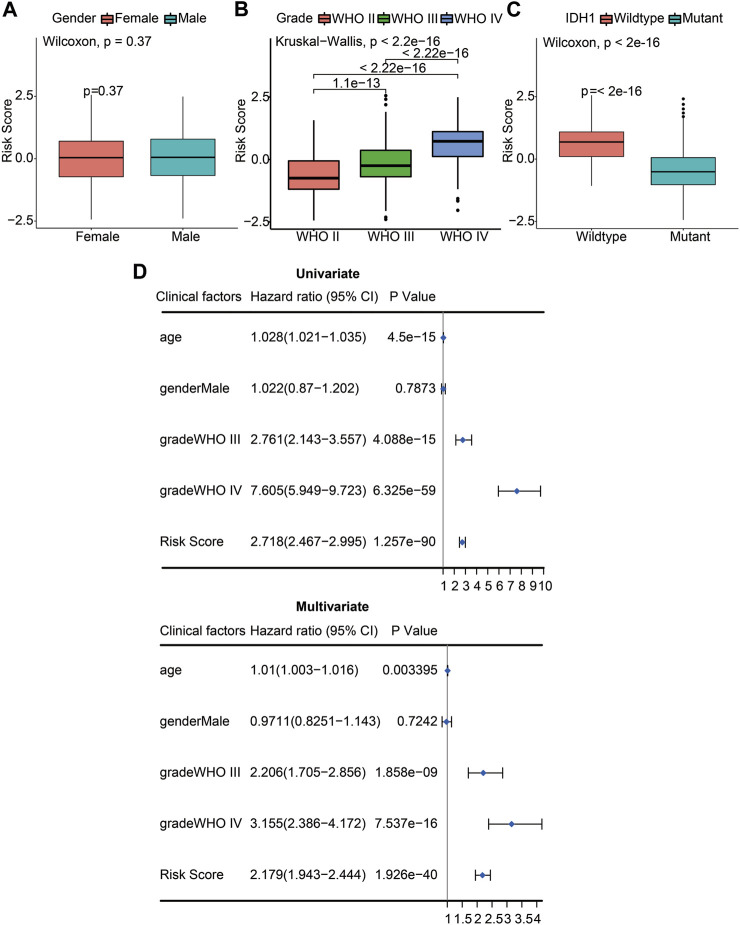
The risk score and WHO stage were independent prognostic factors of gliomas. **(A)** Differences of risk scores between female and male patients (*p* = 0.37). **(B)** Differences of risk scores between pathologic stages including WHO II, WHO III, and WHO IV (*p* <2.2e-16). **(C)** Differences of risk scores between the IDH1 wildtype and mutant glioma patients (*p* <2e-16). **(D)** Univariate Cox (left) and multivariate Cox (right) regression analyses were performed on five prognostic indicators of glioma patients. The blue squares represented the HR, and the short transverse lines represented 95% CI. *p* <0.05 was considered significant.

We used the constructed risk model to predict the risk scores for different subgroups of glioma patients. When comparing the risk scores between female and male patients, there was no statistically significant difference (*p* = 0.37; Figure 5A). The WHO stage is positively associated with poor prognosis in glioma patients. When comparing the risk scores of patients with different WHO stages, including WHO II, WHO III, and WHO IV, the results showed that the risk score of WHO IV had a significant difference compared with WHO III (*p* <0.05) or WHO II (*p* <0.05). Moreover, the risk score of WHO III also had a significant difference with the WHO II risk score (*p* <0.05) determined by the Kruskal–Wallis test. A significant positive association between WHO grades and the risk score is displayed in Figure 5B. We also analyzed the risk scores of the IDH1 mutant and wildtype glioma patients. The results indicated that the IDH1 mutant group had significantly decreased risk scores (*p* <0.05), indicating a better prognosis for patients with IDH1 mutations (Figure 5C). To verify whether the risk scores derived from the prognostic model can be used as an independent prognostic factor, univariate and multivariate Cox regression analyses were performed with the CGGA dataset. From the forest plots, the hazard ratios (HRs) of the risk score were 2.718 (95% confidence interval (CI), 2.467–2.995, *p* <0.05) and 2.179 (95% CI, 1.943–2.444, *p* <0.05), respectively. This result suggests that risk score could be considered an independent prognostic factor for the survival of glioma patients. Meanwhile, WHO grade was also an independent prognostic factor, the HRs of WHO grade III were 2.761 (95% CI, 2.143–3.357, *p* <0.05) and 2.206 (95% CI, 1.705–2.856, *p* <0.05), respectively, and the HRs of WHO grade IV were 7.605 (95% CI, 5.949–9.723, *p* <0.05) and 3.155 (95% CI, 2.386–4.172, *p* <0.05), respectively (Figure 5D). These results confirm that the risk score and WHO staging grades III and IV can be used as independent clinical prognostic factors for patients with gliomas.

### 3.6 Association of infiltrating immune cells with the risk score and prognosis

**FIGURE 6:**
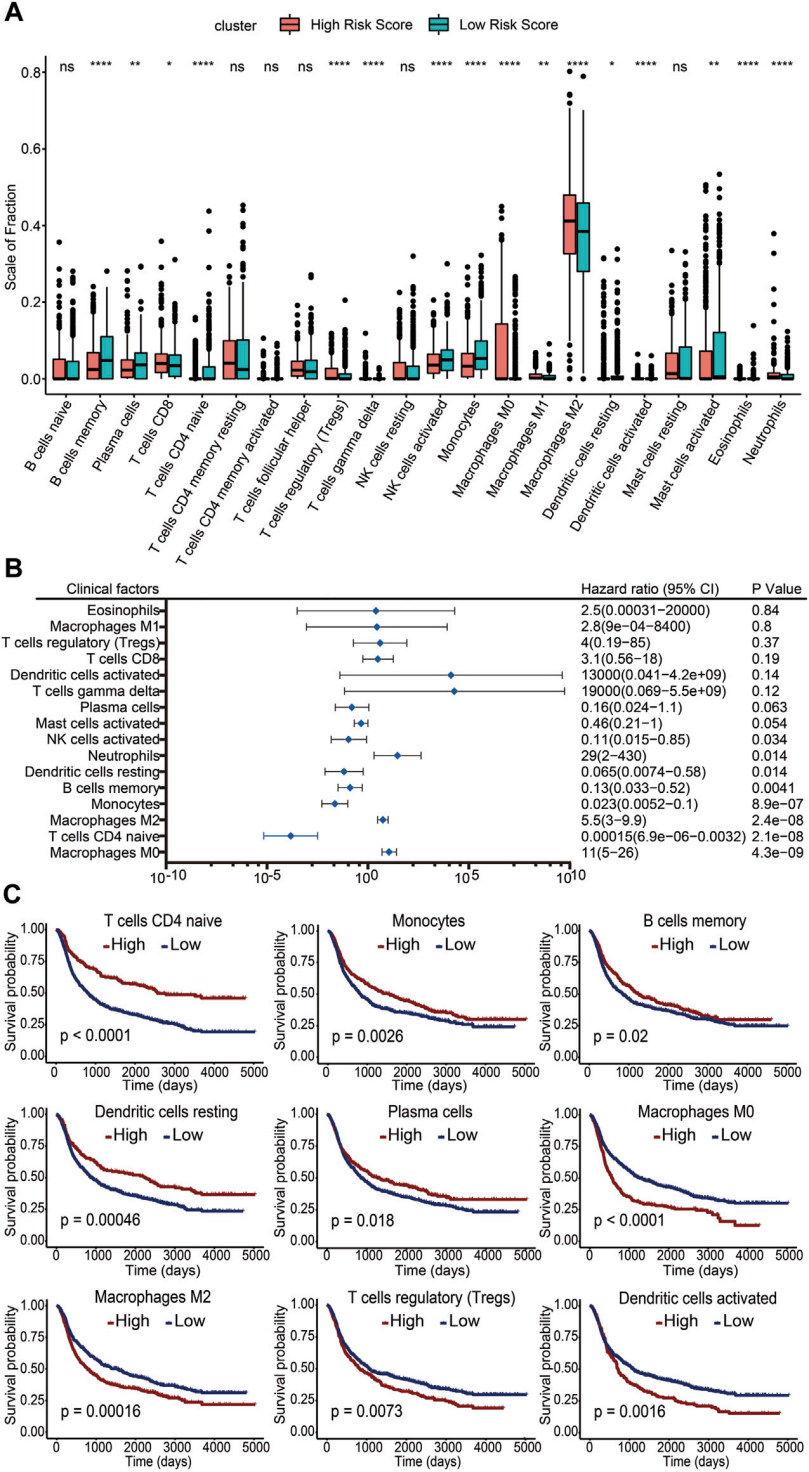
Association of infiltrating immune cells with the risk score and prognosis. **(A)** Level of immune cell infiltration between the high-risk score group and the low-risk score group. ns, no significance. *: *p* <0.05. **: *p* <0.01. ***: *p* <0.001. **(B)** Infiltration levels of 16 immune cell subgroups by univariate Cox regression. **(C)** Kaplan–Meier survival curves of different infiltration levels of immune cells. A *p* value less than 0.05 was considered significant.

To clarify the impact of immune cells on glioma prognosis, we analyzed immune cell infiltration in the TME using the CIBERSORT algorithm and then compared the differences of immune cell presence in gliomas between the high- and low-risk score subgroups. The results showed that infiltration levels of memory B cells, plasma cells, naïve CD4^+^ T cells, activated NK cells, monocytes, resting dendritic cells, activated mast cells, and eosinophils were increased in the low-risk score group. In contrast, CD8^+^ T cells, regulatory T cells (Tregs), γδ T cells, M0 macrophages, M1 macrophages, M2 macrophages, activated dendritic cells, and neutrophils were increased in the high-risk score group (Figure 6A). These results suggested that while the former set of immune cells might be linked to a favorable prognosis, the latter may be associated with a poorer outcome. To further investigate the potential risk of infiltrating immune cells on survival, we performed univariate Cox regression analysis and calculated the HRs with 95% CI. M0 macrophages, M2 macrophages, and neutrophils emerged as independent risk factors for unfavorable glioma prognosis (HR >1; *p* <0.05). Conversely, CD4 naïve T cells, monocytes, memory B cells, resting dendritic cells, and activated NK cells appeared as potential protective factors (HR <1; *p* <0.05) (Figure 6B). Kaplan–Meier survival analysis was further performed in these immune cells. We found pronounced survival probability differences associated with nine immune cell types. Notably, increased levels of memory B cells, resting dendritic cells, monocytes, plasma cells, and naïve CD4 T cells correlated with prolonged survival, while high levels of activated dendritic cells, M0 macrophages, M2 macrophages, and Tregs were linked to shorter survival (Figure 6C). In conclusion, our study underscored the variability in immune cell infiltration across different risk score groups. Crucially, specific immune cell infiltration levels emerged as influential determinants in glioma prognosis.

## 4 Discussion

Glioma is a fatal brain malignancy worldwide with an extremely poor prognosis. Therefore, the construction of predictive models and identification of reliable biomarkers are key requirements for glioma prognosis. In this study, we identified 21 immune-related genes differentially expressed in the TME (*TMSB4X*, *IFNGR2*, *SERPINA3*, *VIM*, *CHGB*, *JUN*, *BMP2*, *TXLNA*, *CDC42*, *HDAC1*, *PSMC2*, *SSTR2*, *IL-13RA2*, *IL17D*, *ADCYAP1R1*, *PAK4*, *PDK1*, *UCN*, *GDF10*, *ARRB1*, and *TMSB15A*), and these genes were significantly associated with the prognosis of glioma patients. Patients with high expression levels of CHGB, BMP2, SSTR2, IL17D, ADCYAP1R1, UCN, GDF10, and ARRB1 had longer overall survival. Among them, IFNGR2, TXLNA, PSMC2, and TMSB15A have not been reported to be associated with gliomas.

We constructed a prognostic risk model based on the 21 immune-related DEGs. The performance of the model was assessed by the ROC curves of the training cohort of CGGA data (AUC = 0.848) and the validation cohort of TCGA data (AUC = 0.846). Our model had a higher specificity and sensitivity than previously reported prognostic models for gliomas (AUC <0.8) (Pagès et al., 2018; Zeng et al., 2019). By analyzing the relationship between the risk score of the constructed prognostic model and overall survival, we found that overall survival was shorter for patients in the high-risk score group. The patients in the high-risk score group were considered to have a poor prognosis. Therefore, the risk score can be used as a molecular indicator of poor prognosis. Previous studies have reported several independent prognostic indicators for gliomas, such as age and WHO grade (Pignatti et al., 2002; Louis et al., 2007; Reuss et al., 2015). In this study, we found a significant positive correlation between clinical WHO grades and the risk score, which was consistent with previous reports (Weller et al., 2013; Khasraw et al., 2014; Glioma through the looking GLASS, 2018). Meanwhile, we found that patients with the *IDH1* gene mutation had lower risk scores (Theeler et al., 2012; Eckel-Passow et al., 2015). In addition, the risk score was validated as an independent prognostic factor for gliomas by univariate and multivariate Cox regression analyses, as well as age and WHO grades.

In this study, GO and KEGG analyses displayed genes enriched for cytokine–cytokine receptor interaction signaling pathways, and single-gene survival analysis showed that immune DEGs for cytokines or receptors, such as *BMP2*, *ARRB1*, *IL17D*, *IL-13RA2*, *ADCYAP1R1*, *IFNGR2*, *UCN*, *SSTR2*, and *GDF10*, were significantly associated with overall survival (Bao et al., 2020). IL-13RA2 is a cell surface receptor that is not significantly expressed in normal brain tissue; however, it is overexpressed in most cancer cells and is currently important in the treatment of brain tumors (Debinski et al., 1999; Kawakami et al., 2004; Brown et al., 2012; Brown et al., 2016). Mantovani A. *et al.* reported that IL-13 can stimulate the activation of M2 macrophages (Mantovani et al., 2002). The presence of M2 in the TME plays a key role in the inflammatory cycle that disrupts adaptive immunity and promotes tumor growth and development (Mantovani et al., 2002). In this study, we found that the infiltration level of M2 was significantly higher in the high-risk score group than in the low-risk score group. Kaplan–Meier survival analysis showed a lower survival probability in glioma patients with a higher level of M2 infiltration. This result suggests that M2 in the TME may be involved in the formation and development of gliomas.

Several studies reported that regulatory T cells interact with intra-tumor antigen-presenting cells (APCs) to promote localization to induce cytotoxic T lymphocyte (CTL) dysfunction and prevent tumor rejection (Bauer et al., 2014; Speiser et al., 2016). APCs mainly consist of dendritic cells and macrophages, with M2 having weak levels of antigen presentation and co-stimulatory capacity (Gordon, 2003; Schmieder et al., 2012). In this study, the infiltration level of activated dendritic cells, M2, and Tregs, was higher in the high-risk score group than in the low-risk score group. Survival analysis showed that patients with higher infiltration levels of activated dendritic cells, M2 and Tregs, had lower survival probability. These results were consistent with those of previous studies (Bauer et al., 2014; Speiser et al., 2016). Thus, the results indicated that the differences in the infiltration levels of immune cells between the high-risk and low-risk score groups may be an important factor contributing to the impact of the risk score on the prognosis of patients with gliomas. However, further investigations are still needed to elucidate the specific mechanisms by which infiltrating immune cells in the TME affect the prognosis of gliomas.

Thomas DA *et al.* investigated the role of TGF-β in tumor evasion of immune surveillance and demonstrated that TGF-β attenuated glioma rejection by inhibiting the cellular expression of key effector molecules, such as granzyme B and IFN-γ (Thomas and Massagué, 2005). We found that immune genes such as BMP2 and GDF10, which belong to the TGF-β family, were differentially expressed between high-risk and low-risk score groups. However, the expression levels of BMP2 and GDF10 were significantly positively correlated with the overall survival in glioma patients, which is inconsistent with the previous findings, suggesting that TGF-β family genes have complex regulatory functions in gliomas. Additionally, we identified that the *JUN* gene was differentially expressed between high-risk score and low-risk score groups. Liu Y *et al.* reported that the JUN/miR-22/HuR regulatory axis played a crucial role in colorectal cancer (CRC) progression (Liu et al., 2018). In CRC cells, the *JUN* gene binds to the USP28 promoter and is involved in KRAS-mediated transcriptional activation to promote CRC formation. However, whether this mechanism occurs in gliomas is unclear and requires further validation.

Nevertheless, our study has some limitations. Although our results had a validation cohort of TCGA to evaluate the performance of the prognostic risk model, the model still needs the support of a large number of clinical samples and clinical data of glioma patients. Moreover, the prognostic risk model was based on RNA-sequencing results from the CGGA database and lacks cellular and animal experiments.

In conclusion, we identified 21 immune-related DEGs that affected prognosis based on immune scores of the TME in gliomas. We also found that the risk score from the 21 genes was an independent risk factor for glioma prognosis, and patients with high risk scores had a poorer prognosis. Furthermore, infiltrating activated dendritic cells, M0 macrophages, M2 macrophages, and Tregs were associated with poor prognosis of gliomas. The immune-associated signature of the glioma microenvironment, including immune-related genes, the risk score, and immune cells significantly associated with prognosis, can be considered new indicators for clinical prognosis assessment of glioma patients.

## Data Availability

The original contributions presented in the study are included in the article/Supplementary Material; further inquiries can be directed to the corresponding authors.

## References

[B1] (2018). Glioma through the looking GLASS: molecular evolution of diffuse gliomas and the glioma longitudinal analysis consortium. Neuro-oncology 20 (7), 873–884. 10.1093/neuonc/noy020 29432615 PMC6280138

[B2] Bao M. H. Lv Q. L. Szeto V. Wong R. Zhu S. Z. Zhang Y. Y. (2020). TRPM2‐AS inhibits the growth, migration, and invasion of gliomas through JNK, c‐Jun, and RGS4. J. Cell. physiology 235 (5), 4594–4604. 10.1002/jcp.29336 31637708

[B3] Bauer C. A. Kim E. Y. Marangoni F. Carrizosa E. Claudio N. M. Mempel T. R. (2014). Dynamic Treg interactions with intratumoral APCs promote local CTL dysfunction. J. Clin. investigation 124 (6), 2425–2440. 10.1172/JCI66375 PMC4089459 24812664

[B4] Brown C. E. Alizadeh D. Starr R. Weng L. Wagner J. R. Naranjo A. (2016). Regression of glioblastoma after chimeric antigen receptor T-cell therapy. N. Engl. J. Med. 375 (26), 2561–2569. 10.1056/NEJMoa1610497 28029927 PMC5390684

[B5] Brown C. E. Starr R. Aguilar B. Shami A. F. Martinez C. D'Apuzzo M. (2012). Stem-like tumor-initiating cells isolated from IL13Rα2 expressing gliomas are targeted and killed by IL13-zetakine-redirected T Cells. Clin. Cancer Res. 18 (8), 2199–2209. 10.1158/1078-0432.CCR-11-1669 22407828 PMC3578382

[B6] Debinski W. Gibo D. M. Hulet S. W. Connor J. R. Gillespie G. Y. (1999). Receptor for interleukin 13 is a marker and therapeutic target for human high-grade gliomas. Clin. Cancer Res. 5 (5), 985–990. 10353730

[B7] Eckel-Passow J. E. Lachance D. H. Molinaro A. M. Walsh K. M. Decker P. A. Sicotte H. (2015). Glioma groups based on 1p/19q, IDH, and TERT promoter mutations in tumors. N. Engl. J. Med. 372 (26), 2499–2508. 10.1056/NEJMoa1407279 26061753 PMC4489704

[B8] Fakih M. Ouyang C. Wang C. Tu T. Y. Gozo M. C. Cho M. (2019). Immune overdrive signature in colorectal tumor subset predicts poor clinical outcome. J. Clin. investigation 129 (10), 4464–4476. 10.1172/JCI127046 PMC6763253 31524634

[B9] Furnari F. B. Fenton T. Bachoo R. M. Mukasa A. Stommel J. M. Stegh A. (2007). Malignant astrocytic glioma: genetics, biology, and paths to treatment. Genes & Dev. 21 (21), 2683–2710. 10.1101/gad.1596707 17974913

[B10] Galon J. Pagès F. Marincola F. M. Thurin M. Trinchieri G. Fox B. A. (2012). The immune score as a new possible approach for the classification of cancer. Springer 10, 1–4. 10.1186/1479-5876-10-1 PMC3269368 22214470

[B11] Gilbert M. R. Dignam J. J. Armstrong T. S. Wefel J. S. Blumenthal D. T. Vogelbaum M. A. (2014). A randomized trial of bevacizumab for newly diagnosed glioblastoma. N. Engl. J. Med. 370 (8), 699–708. 10.1056/NEJMoa1308573 24552317 PMC4201043

[B12] Gordon S. (2003). Alternative activation of macrophages. Nat. Rev. Immunol. 3 (1), 23–35. 10.1038/nri978 12511873

[B13] Hoy A. J. Balaban S. Saunders D. N. (2017). Adipocyte–tumor cell metabolic crosstalk in breast cancer. Trends Mol. Med. 23 (5), 381–392. 10.1016/j.molmed.2017.02.009 28330687

[B14] Hu H. Wang Z. Li M. Zeng F. Wang K. Huang R. (2017). Gene expression and methylation analyses suggest DCTD as a prognostic factor in malignant glioma. Sci. Rep. 7 (1), 11568–11569. 10.1038/s41598-017-11962-y 28912488 PMC5599690

[B15] Iyengar N. M. Gucalp A. Dannenberg A. J. Hudis C. A. (2016). Obesity and cancer mechanisms: tumor microenvironment and inflammation. J. Clin. Oncol. 34 (35), 4270–4276. 10.1200/JCO.2016.67.4283 27903155 PMC5562428

[B16] Jia D. Li S. Li D. Xue H. Yang D. Liu Y. (2018). Mining TCGA database for genes of prognostic value in glioblastoma microenvironment. Aging (Albany NY) 10 (4), 592–605. 10.18632/aging.101415 29676997 PMC5940130

[B17] Kawakami M. Kawakami K. Takahashi S. Abe M. Puri R. K. (2004). Analysis of interleukin-13 receptor alpha2 expression in human pediatric brain tumors. Cancer 101 (5), 1036–1042. 10.1002/cncr.20470 15329913

[B18] Khasraw M. Ameratunga M. S. Grant R. Wheeler H. Pavlakis N. (2014). Antiangiogenic therapy for high-grade glioma. Cochrane Database Syst. Rev. 9, CD008218. 10.1002/14651858.CD008218.pub3 25242542

[B19] Liu Y. Chen X. Cheng R. Yang F. Yu M. Wang C. (2018). The Jun/miR-22/HuR regulatory axis contributes to tumourigenesis in colorectal cancer. Mol. Cancer 17 (1), 11–15. 10.1186/s12943-017-0751-3 29351796 PMC5775639

[B20] Louis D. N. Ohgaki H. Wiestler O. D. Cavenee W. K. Burger P. C. Jouvet A. (2007). The 2007 WHO classification of tumours of the central nervous system. Acta neuropathol. 114, 97–109. 10.1007/s00401-007-0243-4 17618441 PMC1929165

[B21] Mantovani A. Sozzani S. Locati M. Allavena P. Sica A. (2002). Macrophage polarization: tumor-associated macrophages as a paradigm for polarized M2 mononuclear phagocytes. Trends Immunol. 23 (11), 549–555. 10.1016/s1471-4906(02)02302-5 12401408

[B22] Pagès F. Mlecnik B. Marliot F. Bindea G. Ou F-S. Bifulco C. (2018). International validation of the consensus immunoscore for the classification of colon cancer: a prognostic and accuracy study. Lancet 391 (10135), 2128–2139. 10.1016/S0140-6736(18)30789-X 29754777

[B23] Pignatti F. Van Den Bent M. Curran D. Debruyne C. Sylvester R. Therasse P. (2002). Prognostic factors for survival in adult patients with cerebral low-grade glioma. J. Clin. Oncol. 20 (8), 2076–2084. 10.1200/JCO.2002.08.121 11956268

[B24] Reuss D. E. Mamatjan Y. Schrimpf D. Capper D. Hovestadt V. Kratz A. (2015). IDH mutant diffuse and anaplastic astrocytomas have similar age at presentation and little difference in survival: a grading problem for WHO. Acta neuropathol. 129, 867–873. 10.1007/s00401-015-1438-8 25962792 PMC4500039

[B25] Schmieder A. Michel J. Schönhaar K. Goerdt S. Schledzewski K. (2012). Differentiation and gene expression profile of tumor-associated macrophages. Seminars cancer Biol. 2012, 289–297. 10.1016/j.semcancer.2012.02.002 22349514

[B26] Speiser D. E. Ho P-C. Verdeil G. (2016). Regulatory circuits of T cell function in cancer. Nat. Rev. Immunol. 16 (10), 599–611. 10.1038/nri.2016.80 27526640

[B27] Stupp R. Hegi M. E. Mason W. P. Van Den Bent M. J. Taphoorn M. J. Janzer R. C. (2009). Effects of radiotherapy with concomitant and adjuvant temozolomide versus radiotherapy alone on survival in glioblastoma in a randomised phase III study: 5-year analysis of the EORTC-NCIC trial. lancet Oncol. 10 (5), 459–466. 10.1016/S1470-2045(09)70025-7 19269895

[B28] Stupp R. Mason W. P. Van Den Bent M. J. Weller M. Fisher B. Taphoorn M. J. (2005). Radiotherapy plus concomitant and adjuvant temozolomide for glioblastoma. N. Engl. J. Med. 352 (10), 987–996. 10.1056/NEJMoa043330 15758009

[B29] Tan A. C. Ashley D. M. López G. Y. Malinzak M. Friedman H. S. Khasraw M. (2020a). Management of glioblastoma: state of the art and future directions. CA a cancer J. Clin. 70 (4), 299–312. 10.3322/caac.21613 32478924

[B30] Tan Y. Q. Li Y. T. Yan T. F. Xu Y. Liu B. H. Yang J. A. (2020b). Six immune associated genes construct prognostic model evaluate low-grade glioma. Front. Immunol. 11, 606164. 10.3389/fimmu.2020.606164 33408717 PMC7779629

[B31] Theeler B. J. Yung W. A. Fuller G. N. De Groot J. F. (2012). Moving toward molecular classification of diffuse gliomas in adults. Neurology 79 (18), 1917–1926. 10.1212/WNL.0b013e318271f7cb 23109653 PMC3525311

[B32] Thomas D. A. Massagué J. (2005). TGF-β directly targets cytotoxic T cell functions during tumor evasion of immune surveillance. Cancer Cell 8 (5), 369–380. 10.1016/j.ccr.2005.10.012 16286245

[B33] Weller M. Cloughesy T. Perry J. R. Wick W. (2013). Standards of care for treatment of recurrent glioblastoma—Are we there yet? Neuro-oncology 15 (1), 4–27. 10.1093/neuonc/nos273 23136223 PMC3534423

[B34] Wu Q. Li J. Li Z. Sun S. Zhu S. Wang L. (2019). Exosomes from the tumour-adipocyte interplay stimulate beige/brown differentiation and reprogram metabolism in stromal adipocytes to promote tumour progression. J. Exp. Clin. Cancer Res. 38, 223–320. 10.1186/s13046-019-1210-3 31138258 PMC6537177

[B35] Yang S. Liu T. Nan H. Wang Y. Chen H. Zhang X. (2020). Comprehensive analysis of prognostic immune‐related genes in the tumor microenvironment of cutaneous melanoma. J. Cell. physiology 235 (2), 1025–1035. 10.1002/jcp.29018 31240705

[B36] Yi N. Tang Z. Zhang X. Guo B. (2019). BhGLM: bayesian hierarchical GLMs and survival models, with applications to genomics and epidemiology. Bioinformatics 35 (8), 1419–1421. 10.1093/bioinformatics/bty803 30219850 PMC7963076

[B37] Yoshihara K. Shahmoradgoli M. Martínez E. Vegesna R. Kim H. Torres-Garcia W. (2013). Inferring tumour purity and stromal and immune cell admixture from expression data. Nat. Commun. 4 (1), 2612. 10.1038/ncomms3612 24113773 PMC3826632

[B38] Zeng D. Li M. Zhou R. Zhang J. Sun H. Shi M. (2019). Tumor microenvironment characterization in gastric cancer identifies prognostic and immunotherapeutically relevant gene signatures. Cancer Immunol. Res. 7 (5), 737–750. 10.1158/2326-6066.CIR-18-0436 30842092

[B39] Zhang Q. Xiang W. Yi D. Xue B. Wen W. Abdelmaksoud A. (2018). Current status and potential challenges of mesenchymal stem cell-based therapy for malignant gliomas. Stem Cell Res. Ther. 9 (1), 228–229. 10.1186/s13287-018-0977-z 30143053 PMC6109313

