# Supplementary material for: Prognostic risk of immune-associated signature in the microenvironment of brain gliomas
Source: Front Genet. 2023 Oct 6;14:1208651. doi: 10.3389/fgene.2023.1208651 (PMC10587408; doi:10.3389/fgene.2023.1208651)
Supplement: Supplementary file 1 [file DataSheet1.docx]

Supplementary Material

**
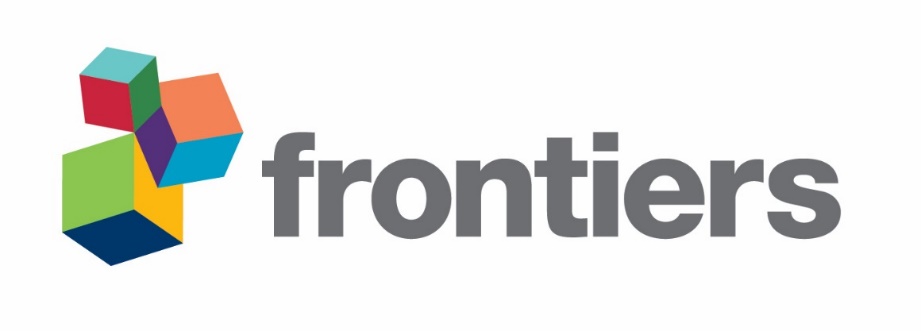
**

**Figure S1** Forestplot of 21 immune-related DEGs by multivariate Cox regression analysis. P value less than 0.05 was thought to be significant.

**Figure S2** Kaplan-Meier survival curves of 21 genes in low-grade glioma (LGG) cohort with p value of log-rank test (**A**, **B**). P value less than 0.05 represented statistically significance.


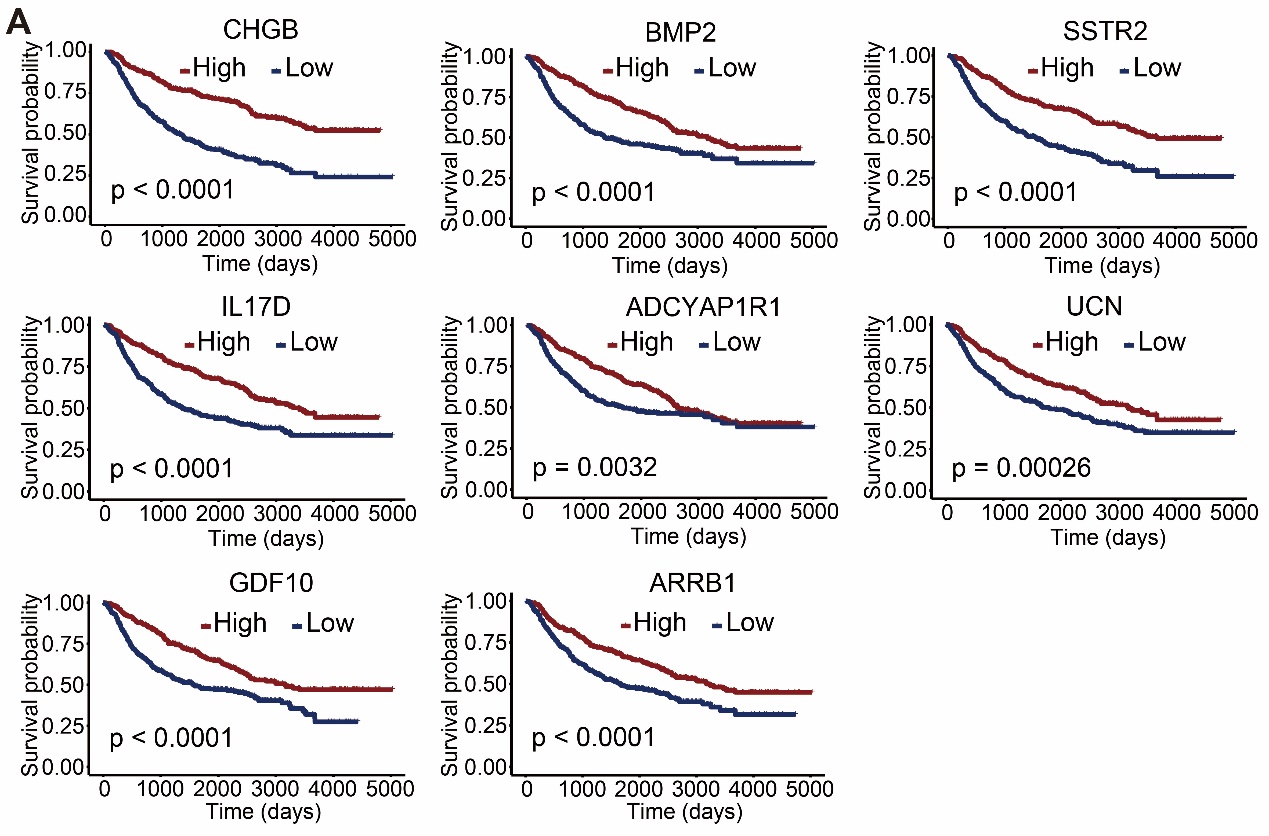


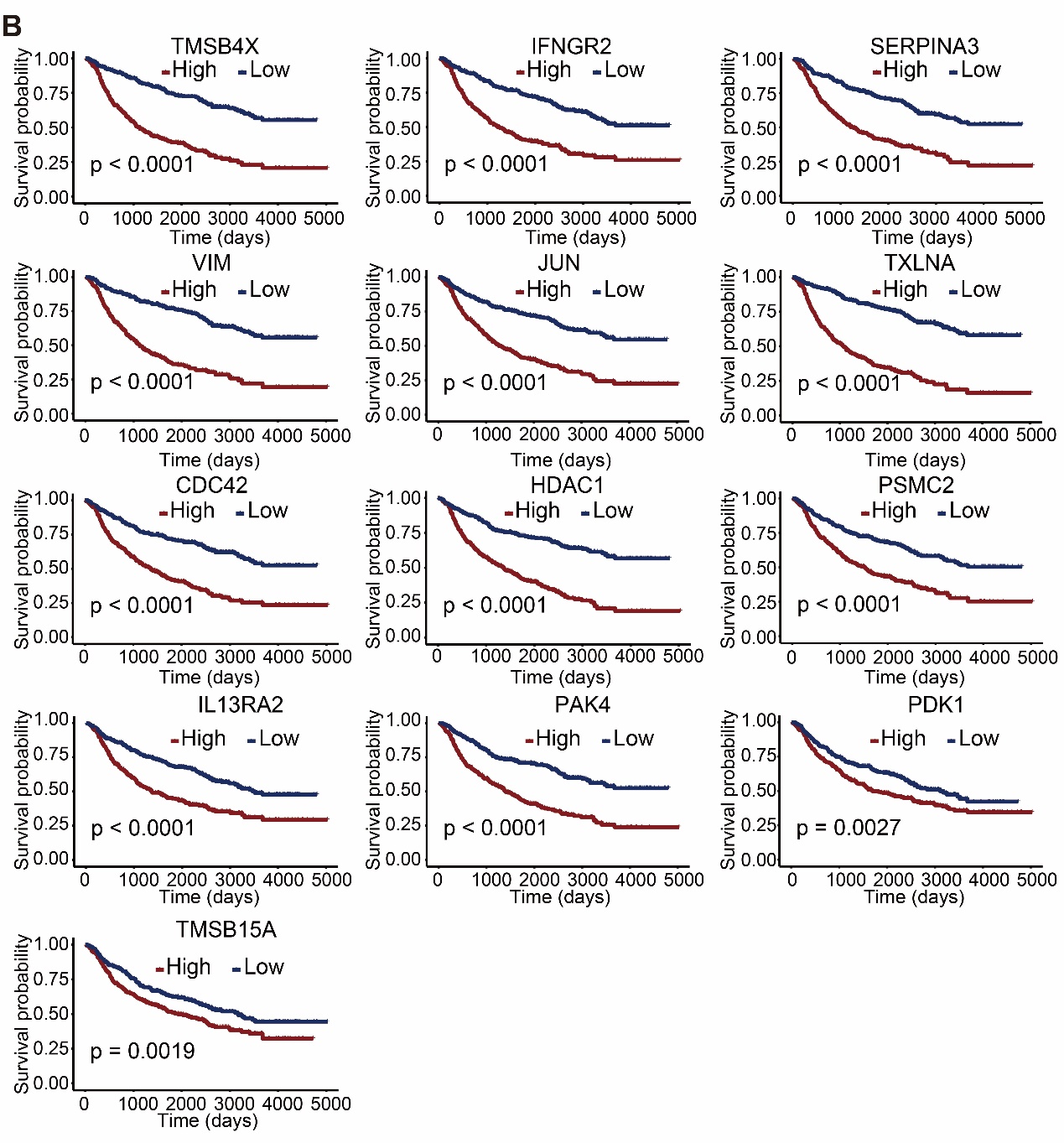


**Figure S3** Kaplan-Meier survival curves of 21 genes in glioblastoma (GBM) cohort with p value of log-rank test (**A**, **B**). P value less than 0.05 represented statistically significance.


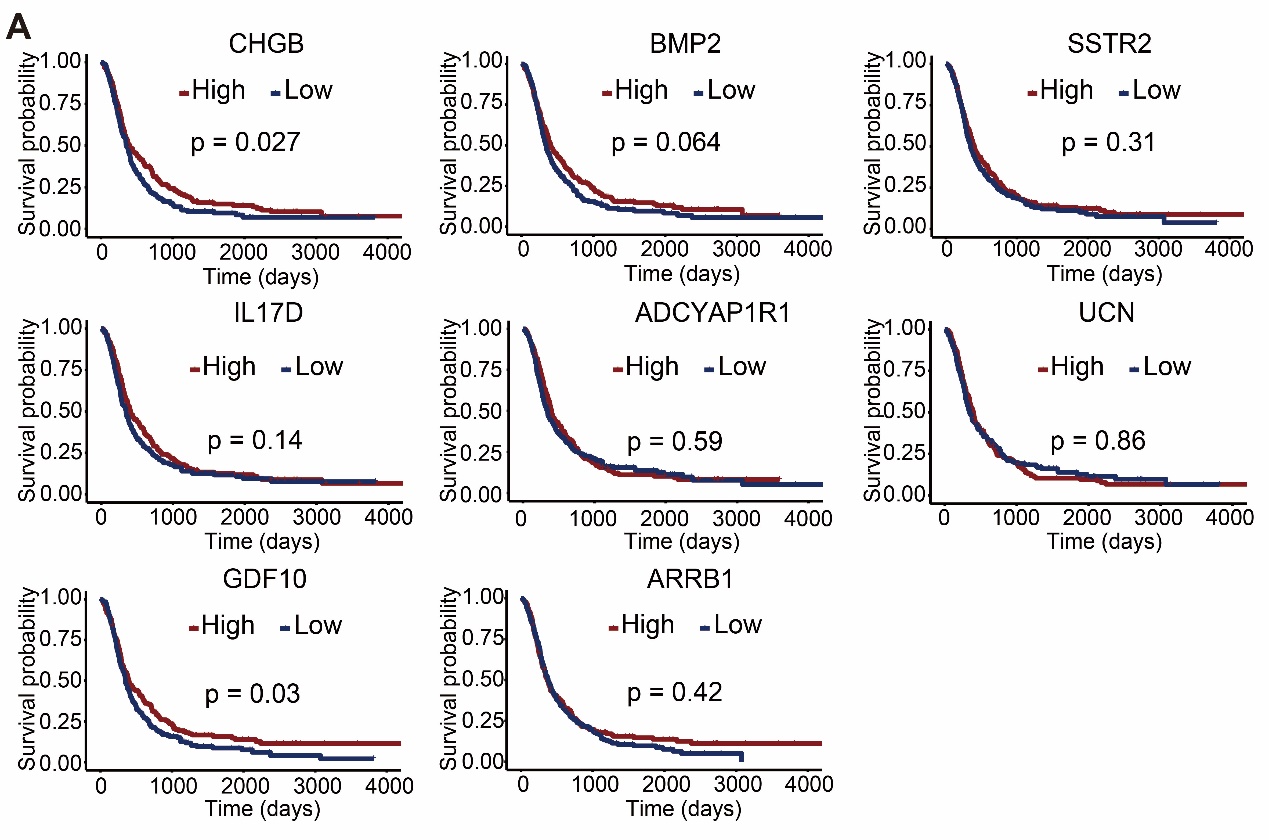


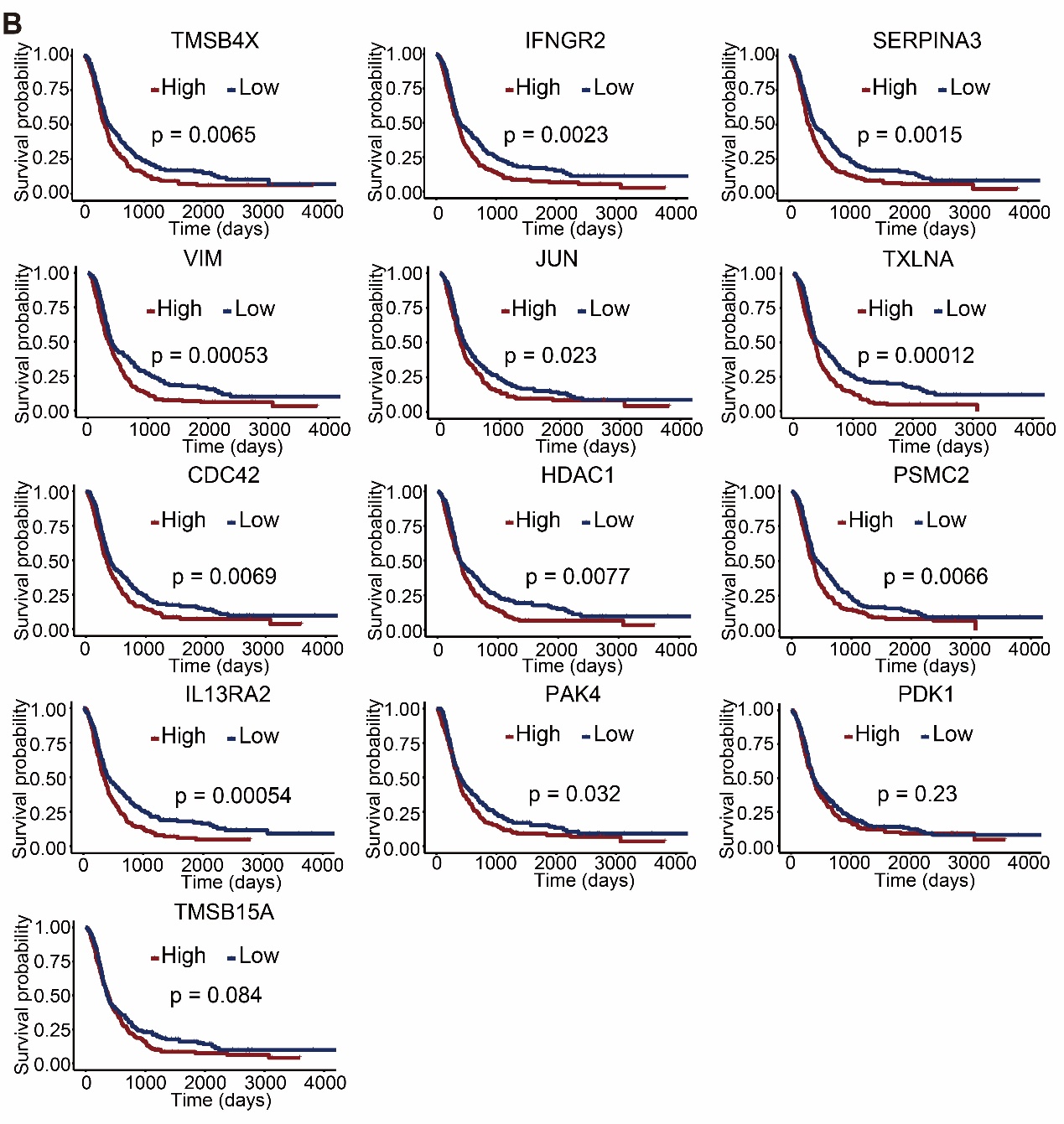


**Figure S4** Nomogram was constructed from clinicopathological parameters and risk score to calculate a patient’s 3-year or 5-year survival probability in CGGA database (**A**) and TCGA database (**B**).


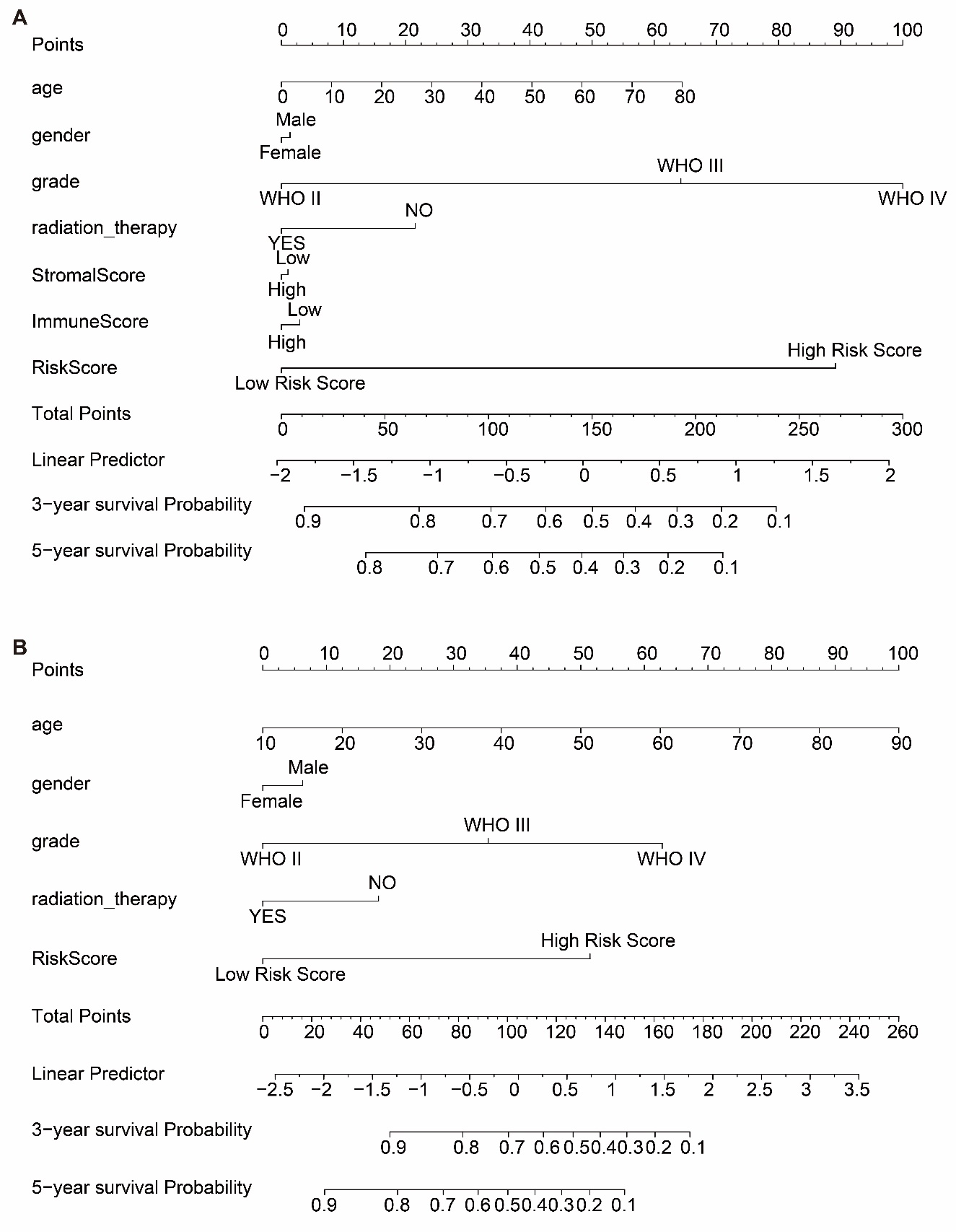


**Figure S5** Decision curve analysis (DCA) and calibration curves for the nomogram in CGGA database (**A**, **B**) and TCGA database (**C**, **D**).


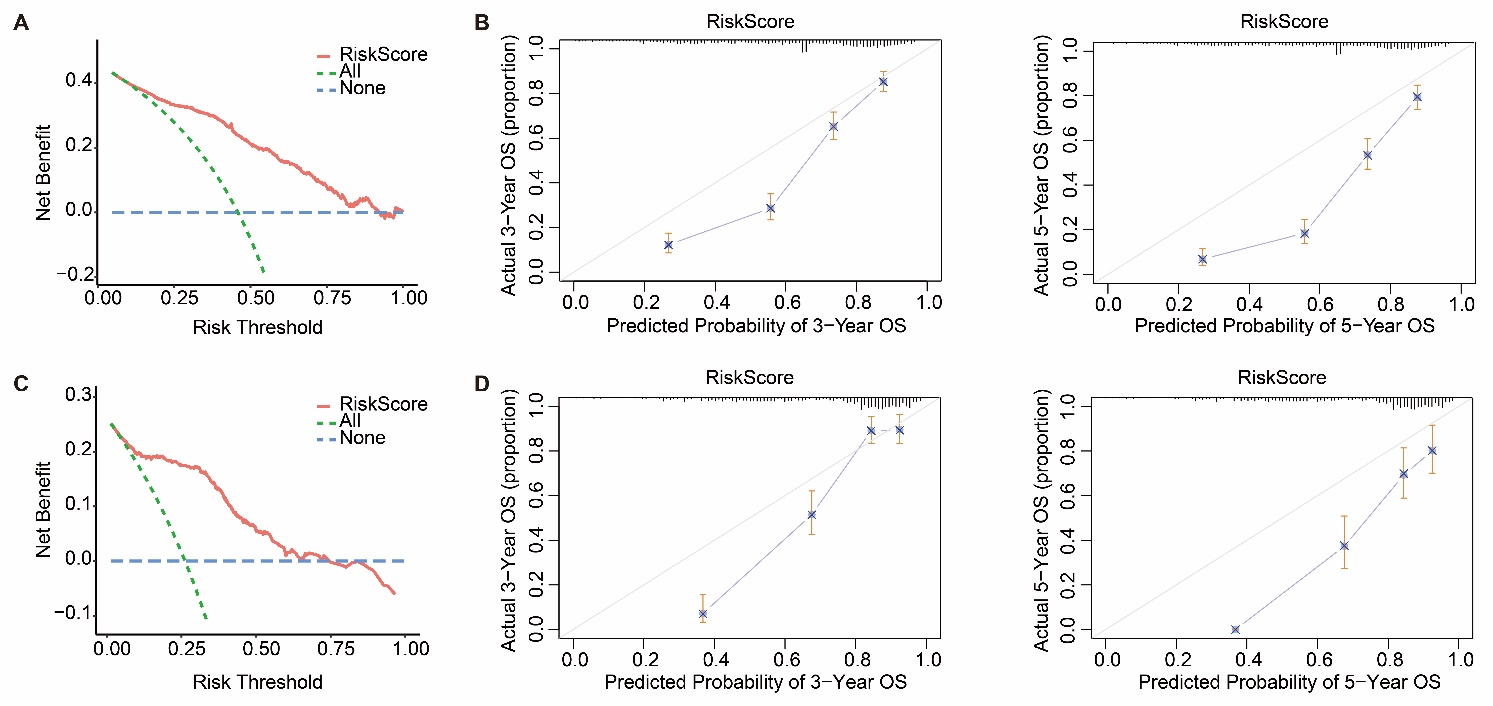


**Table S1** The relationship between stromal scores and different tumor stages. Statistics: Fisher’s exact test.

| **Grade** | **High_stromal score**  **(N=482)** | **Low_stromal score (N=484)** | **P value** |
| --- | --- | --- | --- |
| WHO II | 89 (18.5%) | 181 (37.4%) | <2e-16 |
| WHO III | 129 (26.8%) | 193 (39.9%) |  |
| WHO IV | 264 (54.8%) | 110 (22.7%) |  |

**Table S2** The relationship between immune scores and different tumor stages. Statistics: Fisher’s exact test.

| **Grade** | **High-immune score**  **(N=482)** | **Low-immune score**  **(N=484)** | **P value** |
| --- | --- | --- | --- |
| WHO II | 98 (20.3%) | 172 (35.5%) | <2e-16 |
| WHO III | 139 (28.8%) | 183 (37.8%) |  |
| WHO IV | 245 (50.8%) | 129 (26.7%) |  |
